# Brain Metabolomics in Fragile X-Associated Tremor/Ataxia Syndrome (FXTAS)

**DOI:** 10.3390/cells12172132

**Published:** 2023-08-23

**Authors:** Maria Jimena Salcedo-Arellano, Michael D. Johnson, Yingratana A. McLennan, Ye Hyun Hwang, Pablo Juarez, Erin Lucille McBride, Adriana P. Pantoja, Blythe Durbin-Johnson, Flora Tassone, Randi J. Hagerman, Verónica Martínez-Cerdeño

**Affiliations:** 1 Department of Pathology and Laboratory Medicine, UC Davis School of Medicine, Sacramento, CA 95817, USA; mjsalcedo@ucdavis.edu (M.J.S.-A.); mdajohnson@ucdavis.edu (M.D.J.); yamclennan@ucdavis.edu (Y.A.M.); pjuarez@ucdavis.edu (P.J.); elmcb@ucdavis.edu (E.L.M.); adpantoja@ucdavis.edu (A.P.P.); vmartinezcerdeno@ucdavis.edu (V.M.-C.); 2 Medical Investigation of Neurodevelopmental Disorders (MIND) Institute, University of California Davis, Sacramento, CA 95616, USA; mjsalcedo@ucdavis.edu (M.J.S.-A.); ftassone@ucdavis.edu (F.T.); randijhagerman@ucdavis.edu (R.J.H.); vmartinezcerdeno@ucdavis.edu (V.M.-C.); 3 Institute for Pediatric Regenerative Medicine at Shriners Hospitals for Children Northern California, Sacramento, CA 95817, USA; mjsalcedo@ucdavis.edu (M.J.S.-A.); mdajohnson@ucdavis.edu (M.D.J.); yamclennan@ucdavis.edu (Y.A.M.); pjuarez@ucdavis.edu (P.J.); elmcb@ucdavis.edu (E.L.M.); adpantoja@ucdavis.edu (A.P.P.); vmartinezcerdeno@ucdavis.edu (V.M.-C.); 4 Department of Biochemistry and Molecular Medicine, UC Davis School of Medicine, Sacramento, CA 95817, USA; yehhwang@ucdavis.edu (Y.H.H.); ftassone@ucdavis.edu (F.T.); 5 Division of Biostatistics, Department of Public Health Sciences, UC Davis School of Medicine, Sacramento, CA 95817, USA; bpdurbin@ucdavis.edu; 6 Department of Pediatrics, UC Davis School of Medicine, Sacramento, CA 95817, USA; randijhagerman@ucdavis.edu

**Keywords:** FXTAS, *FMR1*, metabolomics, neurodegeneration

## Abstract

The course of pathophysiological mechanisms involved in fragile X-associated tremor/ataxia syndrome (FXTAS) remains largely unknown. Previous proteomics and metabolomics studies conducted in blood samples collected from *FMR1* premutation carriers with FXTAS reported abnormalities in energy metabolism, and precursors of gluconeogenesis showed significant changes in plasma expression levels in *FMR1* premutation carriers who developed FXTAS. We conducted an analysis of postmortem human brain tissues from 44 donors, 25 brains with FXTAS, and 19 matched controls. We quantified the metabolite relative abundance in the inferior temporal gyrus and the cerebellum using untargeted mass spectrometry (MS)-based metabolomics. We investigated how the metabolite type and abundance relate to the number of cytosine-guanine-guanine (CGG) repeats, to markers of neurodegeneration, and to the symptoms of FXTAS. A metabolomic analysis identified 191 primary metabolites, the data were log-transformed and normalized prior to the analysis, and the relative abundance was compared between the groups. The changes in the relative abundance of a set of metabolites were region-specific with some overlapping results; 22 metabolites showed alterations in the inferior temporal gyrus, while 21 showed differences in the cerebellum. The relative abundance of cytidine was decreased in the inferior temporal gyrus, and a lower abundance was found in the cases with larger CGG expansions; oleamide was significantly decreased in the cerebellum. The abundance of 11 metabolites was influenced by changes in the CGG repeat number. A histological evaluation found an association between the presence of microhemorrhages in the inferior temporal gyrus and a lower abundance of 2,5-dihydroxypyrazine. Our study identified alterations in the metabolites involved in the oxidative-stress response and bioenergetics in the brains of individuals with FXTAS. Significant changes in the abundance of cytidine and oleamide suggest their potential as biomarkers and therapeutic targets for FXTAS.

## 1. Introduction

Fragile X-associated tremor/ataxia syndrome (FXTAS) is a late-onset single-gene neurodegenerative disorder characterized by the following core clinical symptoms: balance problems (ataxia), intention tremor, and cognitive decline. FXTAS is caused by an expanded trinucleotide repeat in the promoter region of the *FMR1* (fragile X messenger ribonucleoprotein 1) gene that codes for a crucial protein, FMRP, important for cognitive development. Not all individuals who are *FMR1* premutation carriers (expansions between 54 and 200 CGG repeats) will go on to develop FXTAS, but the conversion rate increases with age, with the average diagnosis age being around 61 years in males and 68 years in females. Premutation-carrier females have one normal X allele that helps compensate for the expanded one and thus have milder symptoms and a lower FXTAS penetrance rate. Approximately 40% of males and 16% of females develop FXTAS. An added layer of complexity is that some premutation-carrier individuals have mild symptoms; this phenotype is commonly seen in females who were originally thought to be unaffected, while others have symptoms that emerge rapidly and get worse over time.

There is no single and simple test for FXTAS. The sensitivity and specificity of clinical diagnosis methods are low and only effective in patients with significant core symptoms. Magnetic resonance imaging of the brain supports the diagnosis of FXTAS; however, white matter hyperintensities in the middle cerebellar peduncles (MCP sign), a major radiological criterion, are seen in approximately 60% of males and in <13% of females. The pathological examination of postmortem brain is widely accepted as the final diagnostic method of FXTAS by confirming eosinophilic-ubiquitin-positive intranuclear inclusions [1,2]. Like other diseases, an effective drug treatment may be needed before the onset of clinical symptoms. In this context, metabolomics can be a useful tool to detect altered metabolites that are closely associated with FXTAS pathogenesis. Similar to most neurodegenerative disorders with no known cure, there is an urgent need for biomarkers that can facilitate the development, diagnosis, severity, prognosis, and treatment of FXTAS.

Primary metabolites, products of the metabolism that are directly involved in cell maintenance, growth, and reproduction, are emerging substances that can be used as potential disease biomarkers. An imbalance of metabolic homeostasis is a precursor for disease, so the assessment of changes in the metabolome through metabolomics analysis has become an important application for understanding the complexity of a disease phenotype and for discovering novel therapeutic targets. Previous metabolomics studies conducted in human plasma and brain tissue from animal models showed metabolic differences between *FMR1* premutation with and without FXTAS and controls [3,4,5,6].

Here, we report the metabolomic profile of postmortem brain tissues from patients who died from FXTAS and report the pathways potentially affected by FXTAS pathophysiology [7]. We employed an untargeted metabolomics approach using a combination of gas chromatography coupled with mass spectrometry (GC-MS) for a global metabolic profile of the postmortem human brain cortex and cerebellum (CB) from individuals with FXTAS. This approach accurately quantified 513 primary metabolites, with 191 being identified, from 44 tissue samples harvested from the inferior temporal gyrus (ITG) and CB. We performed a pathway enrichment analysis to detect altered biological processes and a receiver operator characteristic (ROC) curve analysis on a selected list of metabolites to assess for their discriminatory potential. These findings can potentially help to identify the key predictive biomarkers of FXTAS and expand the basic knowledge of the metabolome. Understanding the metabolic processes associated with core clinical symptoms in FXTAS may aid in uncovering biochemical pathways associated with different FXTAS phenotypes.

## 2. Materials and Methods

### 2.1. Participants

Twenty-five brains with FXTAS and nineteen matched controls for age and sex were included in this study (Table 1). FXTAS cases were obtained from the FXS/FXTAS brain repository at UC Davis (a node of the Hispanic-American Brain Bank for Neurodevelopmental Disorders—CENE) [8]. All donors gave written informed consent for a brain autopsy and the use of the material and clinical information for research purposes. Control brain tissues were obtained from the NIH NeuroBioBank and acquired from subjects without any significant neurological history or the *FMR1* premutation. FXTAS cases were advanced in the disease progression and had a variable clinical presentation. FXTAS diagnosis was confirmed with the presence of the *FMR1* premutation and postmortem ubiquitin-positive intranuclear inclusions in brain cells.

### 2.2. GC-MS Sample Preparation and Metabolite Profiling

Five hundred milligrams (0.5 g) of fresh frozen brain tissue were sampled from the ITG (Brodmann area 20) and the CB. Samples were stored at −80 degrees Celsius prior to analysis. Metabolite relative abundance was conducted by the West Coast Metabolomics Center using GC-MS quantification. Samples were extracted using 1 mL of 3:3:2 ACN:IPA:H2O (*v*/*v*/*v*). Half of the sample was dried to completeness and then derivatized using 10 µL of 40 mg/mL methoxyamine in pyridine. The samples were shaken at 30 °C for 1.5 h. Then, 91 µL of *N*-methyl-*N*-(trimethylsilyl) trifluroacetamide (MSTFA) + fatty acid methyl esters (FAMEs) were added to each sample, and they were shaken at 37 °C for 0.5 h to finish derivatization. The samples were then vialed, capped, and injected onto the instrument. Data were acquired using the chromatographic parameters detailed in Fiehn O. et al. [9]. Metabolite profiling was performed by an ALEX-CIS GC TOF MS system from the Gerstel corporation (Linthicum, MD, USA). Chromatographic parameters were as follows: column, Restek corporation Rtx-5Sil MS (30 m length × 0.25 mm internal diameter with 0.25 µm film made of 95% dimethyl/5%diphenylpolysiloxane); mobile phase, helium; column temperature, between 50–330 °C; flow rate, 1 mL min^−1^; injection volume, 0.5 µL; injection, 25 splitless time into a multi-baffled glass liner; injection temperature, 50 °C ramped to 250 °C at 12 °C s^−1^. The oven temperature parameters were as follows: 50 °C for 1 min, then ramped at 20 °C min^−1^ to 330 °C and held constant for 5 min. The mass spectrometry parameters were as follows: a Leco Pegasus IV mass spectrometer (St. Joseph, MI, USA) was used with a unit mass resolution at 17 spectra s^−1^ from 80–500 Da at a −70 eV ionization energy and an 1800 V detector voltage with a 230 °C transfer line and a 250 °C ion source. Data analysis was achieved using ChromaTOF vs. 2.32 (St. Joseph, MI, USA). Signal intensities were reported as peak heights using the unique ion as default. Raw data were submitted to BinBase (West Coast Metabolomics Center, Davis, CA, USA). Data were normalized using sum normalization after BinBase data processing during data curation. A series of 13 FAMEs were used as internal standards for the retention index and QC during acquisition to control for batch variation. All metabolomics data generated, in both raw and processed formats, and total ion chromatograms are available at the Metabolomics Workbench www.metabolomicsworkbench.org (accessed on 22 August 2023).

### 2.3. CGG Repeat Length

CGG allele repeat size was obtained from DNA samples isolated from 3 mL of whole blood using the standard procedure (Qiagen, Valencia, CA, USA) and assessed through polymerase chain reaction (PCR) and Southern blot analysis as previously described [10,11]. PCR used specific *FMR1 primers*, and PCR products were visualized by capillary electrophoresis and analyzed with the Peak Scanner Software 2.0 (Thermo Fisher Scientific, Waltham, MA, USA).

### 2.4. Markers of Neurodegeneration

We analyzed the presence/absence of amyloid beta plaques, tau aggregates (neurofibrillary tangles and neuritic plaques), and microhemorrhages, all of which are markers of neurodegenerative processes in the brain. Emerging evidence points to the co-existence of Alzheimer’s-type pathology in the brain cortex and the presence of microhemorrhages in the cerebellum as possible aggravating factors for the rate of disease progression in FXTAS [12,13,14]. Fixed samples from the ITG (Brodmann area 20) and CB were immersed in 30% sucrose and embedded in an optimal cutting temperature compound. Afterwards, each sample block was cut on the cryostat at a 14 μm thickness. We incubated sections in DIVA for 8 minutes at 110 °C followed by 3% hydrogen peroxide, permeabilized and blocked them in a tris-buffered saline (TBS) solution containing Triton and donkey serum for 1 hour (75% TBS, 15% Triton, 10% serum), and incubated them with the following primary antibodies overnight at 4 °C in a dark and humid box: rabbit anti-ubiquitin (1:150; Dako, Glostrup, Denmark), polyclonal rabbit anti-β amyloid 1–42 (1:500; Abcam, Cambridge, UK), and mouse monoclonal anti-phosphorylation clone AT8 tau (1:200, Invitrogen, Waltham, MA, USA). On day 2, sections were incubated with biotinylated secondary antibody (1:150, Jackson ImmunoResearch, West Grove, PA, USA) for 1 hour, incubated in a Vectastain ABC kit (Vector Labs, Burlingame, CA, USA) for 2 h, developed in a DAB kit (Vector Labs, Burlingame, CA, USA), dehydrated with alcohols, cleared in xylene, and cover slipped. Immunochemistry was used to evaluate the presence/absence of neurofibrillary tangles, neuritic plaques, and FXTAS intranuclear inclusions. Hematoxylin- and eosin-stained tissue was evaluated for the presence of microhemorrhages, defined as small (<10 mm), cortical/subcortical perivascular hemorrhages.

### 2.5. Symptoms of FXTAS

We extracted clinical data from available medical histories from FXTAS cases. We studied the association between changes in metabolite abundance in postmortem brain (ITG and CB) and the severity of core clinical symptoms (absent/mild vs. moderate/severe) per last medical report based on the treating physician’s perception during neurological evaluation. FXTAS stages (stages of physical disability defined by the consortium) were as follows: (1) subtle or questionable tremor and/or balance problems; (2) minor tremor and/or balance problems with minimal interference in activities of daily living (ADL); (3) moderate tremor and/or balance problems with significant interference in ADL; (4) severe tremor and/or balance problems with the need to use a cane or walker; (5) daily use of a wheelchair; and (6) bedridden [15]. Scores from standardized clinical or research assessments were also used when available (Supplementary Materials Table S3).

### 2.6. Statistical Methods

#### 2.6.1. Differential Metabolite Abundance Analyses

Cases and controls were frequency matched by age and sex. Metabolomics data were log transformed and cyclic loess were normalized [16] prior to analysis. Differential metabolite abundance analyses were conducted using the Bioconductor package limma [17] version 3.52.2. Each brain region was analyzed separately. Models used in limma were adjusted for batch and subject age by including these as covariates (postmortem interval could not be controlled for as it was missing for several subjects but had a comparable distribution in cases and controls). Analyses were conducted using R version 4.2.1 (22 November 2022) [18]. Multiple comparisons were accounted for using the Benjamini–Hochberg false discovery rate (FDR adj. *p*-value). Metabolites were considered significant when the adj. *p*-value was calculated from the FDR < 0.05, and metabolites with adj. *p*-value < 0.08 were considered trending. Metabolites with raw *p*-values < 0.01 and adj. *p*-values < 0.2 were considered to have a possible association with changes in the number of CGG repeats. Volcano plots of metabolite log2FC and −log10 (*p*-value) values were also generated to identify metabolites of interest, with metabolites considered to be differentially expressed if they exhibited a *p*-value < 0.05.

#### 2.6.2. Enrichment and Pathway Analyses

The differentially expressed metabolites from the comparative analyses of FXTAS versus control groups were subjected to enrichment analysis using the R package clusterProfiler [19], version 4.4.4, which implements the algorithm from GSEA [20]. This analysis is sensitive to identifying small but consistent changes among metabolites involved in the same biological pathway. Pathways were considered significant when the adj. *p*-value calculated from the enrichment analysis was <0.05 and trending when adj. *p*-value 0.05 < 0.08.

#### 2.6.3. Receiver Operator Characteristic (ROC) Curve Analysis—Area under the Curve (AUC)

The ROC curve is the most popular graphical tool for evaluating the discriminatory power of a biomarker. To postulate potential predictive biomarkers of FXTAS, we tested the metabolites with a raw *p*-value < 0.05 in the differential metabolite abundance analysis and those metabolites found to be upregulated/downregulated in the volcano plot analysis for ITG and CB. A value of 0.5 for AUC suggested that the metabolite being tested had no discriminatory ability between FXTAS cases and controls. AUC values between 0.5–0.7 had poor discrimination, 0.7–0.8 had acceptable discrimination, 0.8–0.9 had excellent discrimination, and >0.9 had outstanding discrimination. The AUC values reported are in-sample values.

## 3. Results

A metabolomics dataset was generated from the frozen postmortem brain samples from FXTAS cases and age-/sex-matched controls. The untargeted analysis of the primary metabolites demonstrated significant alterations in the FXTAS brains. A complete list of the identified metabolites is available in the Supplementary Materials. A description of the key outcomes is presented below.

### 3.1. Participants

The age of the FXTAS cases ranged from 67 to 93 years (mean 80.8 years). The control cases ranged in age from 62 to 97 years (mean 80.5 years). The average CGG repeat number in the FXTAS group was 82 ± 15.7 (mean ± SD), and in the controls, it was 30.6 ± 4.8. A total of 100% of the cases (*n* = 44) were white. The age range for the onset of symptoms of FXTAS was 50–81 years (Table 1). Most of the FXTAS cases presented with ataxia as the first clinical manifestation, and 36% of the cases had additional neurological diagnoses (Supplementary Materials Table S3).

### 3.2. Primary Metabolite Profile in the Brains with FXTAS

**Figure 1:**
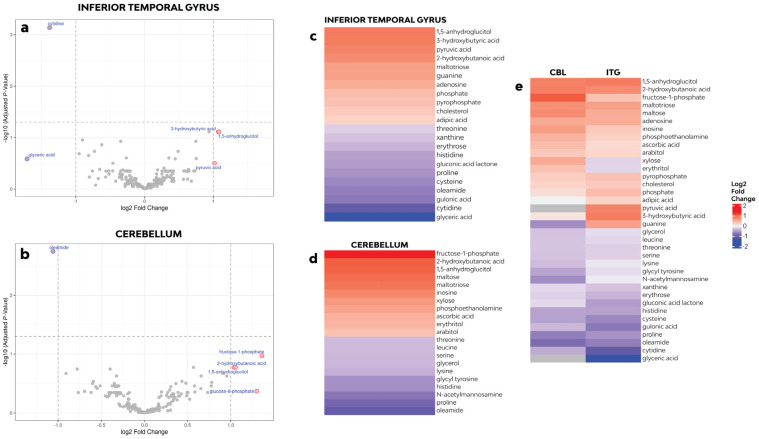
(**a**,**b**) Volcano plots for differential metabolite abundance analysis of case versus control in ITG and CB. The *x*-axis shows the logFC for case/control, and the *y*-axis shows −log10 of the false discovery rate adjusted *p*-value. Metabolites with an absolute logFC greater than 1 are shown in pink and labeled with their BinBase name. The dashed horizontal line shows an adjusted *p*-value of 0.05. The dashed vertical lines show log2 fold changes of −1 and 1. A metabolite was said to be differential if *p*-value < 0.05 and logFC > 1. The purple dots in the figure represent downregulated differentially expressed metabolites, the red dots represent upregulated differentially expressed metabolites, and the gray dots represent metabolites detected but that were not significantly different. (**c**–**e**) Heatmaps of altered metabolites. Heatmaps were created using the Bioconductor package ComplexHeatmap, version 2.12.0. Red indicates high and purple indicates low abundance of the metabolite relative to the median.

The differential metabolite abundance analysis detected 513 primary metabolites, and 191 were identified (Supplementary Materials Tables S1 and S2). The changes in the metabolite relative abundance were region-specific. A total of 22 metabolites showed changes between groups (**raw** *p*-value < 0.05) in ITG. Cytidine abundance, a pyrimidine nucleoside, was lower in the FXTAS cases—log2 fold change (logFC): −1.38, adj. *p*-value 0.0007. In addition, 3-hydroxybutyric acid (logFC: 1.07, adj. *p*-value 0.0772), 2-hydroxybutanoic acid (logFC: 0.93, adj. *p*-value 0.0772), and 1,5-anhydroglucitol (logFC: 1.08, adj. *p*-value 0.0772), metabolites involved in energy metabolism, showed a trending higher abundance. CB showed changes in 21 metabolites (**raw** *p*-value < 0.05), and the relative abundance of oleamide, a fatty acid, was significantly lower (logFC: −1.06, adj. *p*-value 0.0018) (Table 2). Significance based on a volcano plot analysis showed the abundance of 3-hydrobutyric acid, 1,5-anhydroglucitol, and pyruvic acid to be upregulated; cytidine and glyceric acid were downregulated in the ITG of FXTAS cases compared to the controls. In CB, fructose-1-phosphate, 1,5-anhydroglucitol, 2-hydroxybutanoic acid, and glucose-6-phosphate were upregulated, while oleamide was observed to be downregulated, as shown in Figure 1a,b.

### 3.3. KEGG Enrichment Analysis

The enrichment of the KEGG pathway analysis in the ITG revealed that the most relevant downregulated pathways were related to the dysregulation of identified amino acids in FXTAS vs. controls: (i) protein digestion and absorption (hsa04974), (ii) aminoacyl-tRNA biosynthesis (hsa00970), and (iii) D-amino acid metabolism (hsa00470). Similar downregulation of (i) protein digestion and absorption (hsa04974) and (ii) D-amino acid metabolism (hsa00470) were noted in CB (Table 3). The enrichment analysis did not show any distinctive upregulated pathways.

### 3.4. Relationship between CGG Expansion and Metabolite Abundance

**Figure 2:**
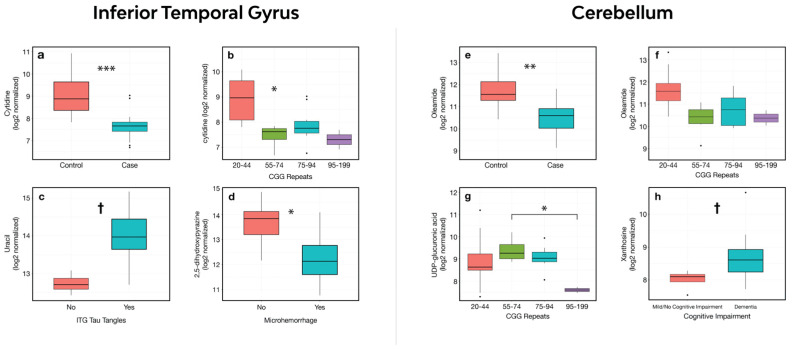
(**a**) Cytidine’s differential abundance cases/controls. (**b**) Cytidine abundance was significantly lower in the 55–74 CGG range group compared to controls in the ITG. (**c**) Uracil was increased, with trending significance, in the ITG of subjects with identified neurofibrillary tangles. (**d**) 2,5-dihydroxypyrazine was significantly decreased in cases with microhemorrhages in the ITG. In cerebellum: (**e**) Oleamide abundance was lower in FXTAS cases compared to controls. (**f**) Differences in relative abundance (raw *p* < 0.001) were found in the 55–74 and 75–94 CGG range groups. Significance was not achieved after multiple testing adjustment. (**g**) Significantly lower abundance of UDP-glucuronic acid in cases with larger expansions > 95 CGG repeats when compared to cases with 55–94 CGG repeats. (**h**) Xanthosine was increased, with trending significance, in the CB of cases with dementia diagnosis per medical history. * adj. *p*-value < 0.05, ** adj. *p*-value < 0.01, *** adj. *p*-value < 0.001, † raw *p*-value < 0.0015.

Forty-six metabolites had abundance changes associated with CGG repeat expansions (raw *p*-value < 0.05), eleven of which showed a possible correlation (raw *p*-value < 0.01 and adj. *p*-value < 0.2) between the relative abundance and the number of CGG repeats in ITG. Cytidine showed a significant decrease in abundance with smaller CGG expansions (55–74 repeats). Cases with larger expansions showed a larger logFC change; however, significance was not achieved after a multiple comparisons adjustment (logFC −1.59; adj. *p*-value 0.03 (55–74 vs. 20–44 repeats); logFC −1.26; adj. *p*-value 0.06 (75–94 vs. 20–44 repeats); logFC −1.82; adj. *p*-value 0.14 (75–94 vs. 95–120 repeats)) (Figure 2a,b). Five metabolites exhibited increases in abundance with increases in the CGG repeat number (which we refer to here as a “positive correlation”, although CGG was categorized in the analysis); 2-hydroxybutanoic acid showed a trending difference (adj. *p*-value 0.054) between cases with larger CGG expansions and controls (Supplementary Materials Figure S1), while adenosine, adipic acid, guanine, and pyrophosphate had possible positive correlations. Six metabolites showed a possible negative correlation: histidine, cytidine, asparagine, xanthine, proline, and heptadecanoic acid. In the CB, 42 metabolites had abundance changes; oleamide and fructose-1-phosphate presented a possible correlation (Supplementary Materials Figure S1). Further analysis of the cases showed a significant decrease in the abundance of UDP-glucuronic acid (logFC: −1.66, adj. *p*-value 0.04)—a sugar made from UDP-glucose using NAD+ as cofactor and used in the synthesis of polysaccharides and ascorbic acid—in cases with larger expansions (>95 CGG repeats) when compared to smaller expansions in the premutation range (Figure 2g). Conflicting results were found in the plasma samples from *FMR1* premutation carriers, with a positive correlation between the relative abundance of proline and UDP-glucuronic acid, and in those with larger CGG expansions [21]. Oleamide, on the other hand, had a negative correlation with the number of CGG repeats in the plasma from premutation carriers with and without FXTAS, which was in agreement with our results in the postmortem brain samples (Figure 2f). There is a possibility that the lack of significance can be attributed to the limited number of FXTAS cases included in the small expansion (CGG 55–74, *n* = 7) and large expansion (CGG > 95, *n* = 4) groups during the analysis.

### 3.5. ROC Curve Analysis

**Figure 3:**
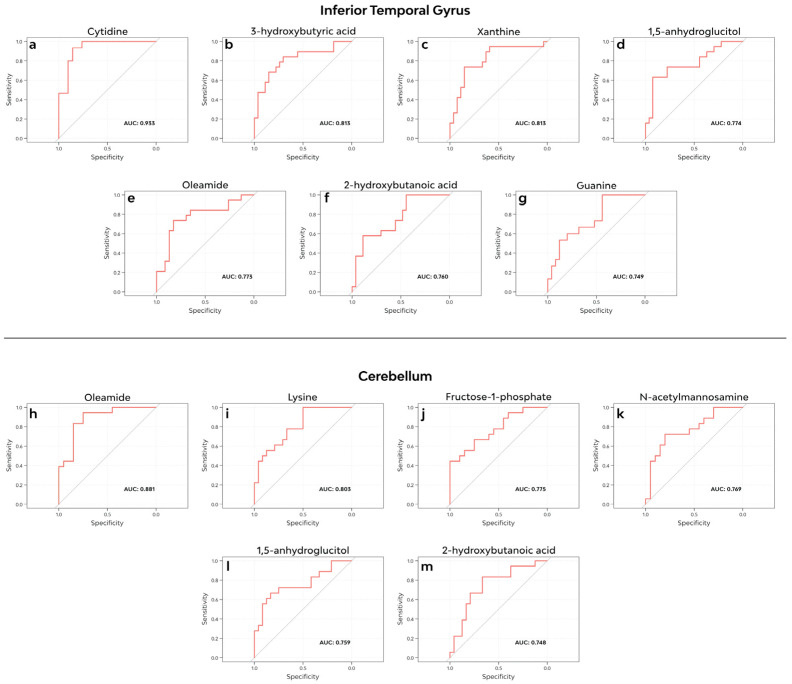
ROC analysis. (**a**–**g**) Metabolites with discriminatory potential between FXTAS and controls in the ITG. (**h**–**m**) Metabolites with discriminatory potential between FXTAS and controls in the CB. AUC less than or equal to 0.5 = no discriminatory potential, AUC 0.5–0.7 = poor discriminatory potential, AUC 0.7–0.8 = acceptable discriminatory potential, AUC 0.8–0.9 = excellent discriminatory potential, and AUC > 0.9 = outstanding discriminatory potential. AUC values reported are in-sample values.

Based on our results from the differential metabolite abundance analyis and volcano plot analysis, we tested 22 metabolites in the ITG and 21 in the CB (see Table 2) with raw *p*-values < 0.05 for their discriminatory potential between the FXTAS cases and controls. We found cytidine to have outstanding discriminatory potential in the ITG, followed by 3-hydroxybutyric acid and xanthine with excellent discriminatory potential and 1,5-anhydroglucitol, oleamide, 2-hydroxybutanoic acid, and guanine with acceptable discriminatory potential. In the CB, oleamide and lysine had excellent discriminatory potential, while fructose-1-phosphate, *N*-acetylmannosamine 1,5-anhydroglucitol, and 2-hydroxybutanoic acid had acceptable discriminatory potential. The remaining tested metabolites had no/poor discriminatory potential for FXTAS vs. controls. (See Figure 3).

### 3.6. Association between Changes in Metabolite Abundance and Markers of Neurodegeneration

We identified possible associations between markers of neurodegeneration (presence/absence) in the ITG of FXTAS cases and changes in metabolite abundance. Uracil was increased by 1.30 logFC (raw *p*-value 0.0007, adj. *p*-value 0.121) in the cases with neurofibrillary tangles (81%, *n* = 18), and 2,5-dihydroxypyrazine was significantly decreased by −1.61 logFC (adj. *p*-value 0.047) (Figure 2c,d) in the cases with microhemorrhages (50%, *n* = 11). Our group previously reported that about 50–60% of FXTAS cases present with microhemorrhages in the white matter of the cerebral and cerebellar cortices [14].

### 3.7. Association between Changes in Metabolite Abundance and Clinical Symptoms

The abundance of xanthosine, a purine nucleoside, was increased by 2.2 logFC with trending significance (adj. *p*-value 0.061) in the CB of the FXTAS cases with a clinical diagnosis of dementia vs. those with no/mild cognitive decline per medical history (Figure 2h). No other association was found between the studied symptoms of FXTAS and the changes in metabolite abundance in the ITG and CB.

## 4. Discussion

Our analysis generated untargeted metabolomics data in postmortem brains with FXTAS. The metabolomics analysis identified disease-related metabolic changes. These data are the starting point to map the metabolic trajectory leading to disease onset and progression. We identified a set of altered metabolites, enhanced pathways, and potential biomarkers of disease at the later stages of FXTAS progression. All the cases evaluated had an advanced clinical involvement. In this section, we discuss our findings, suggesting remarkable metabolic abnormalities in the pathways involved in cellular membrane synthesis, response to oxidative stress, and energy utilization.

### 4.1. Pathways Involved in Neuronal Membrane Synthesis Are Affected in FXTAS

Cytidine’s relative abundance within the ITG was lower (logFC −1.38, adj. *p*-value 0.0007) and showed an optimal discriminatory power between the FXTAS cases and controls (Figure 3a). Its abundance decreased remarkably in the FXTAS cases with larger CGG expansions when compared to the controls with normal (20–44) CGG repeat numbers (Figure 2b). Cytidine is a pyrimidine nucleoside comprising a cytosine nucleic base and ribose sugar and is acquired through dietary sources. Cytidine is a substrate in the production of critical membrane phospholipids, phosphatidylcholine, and phosphatidylethanolamine and is also a precursor in pyrimidine nucleotide synthesis concerning uracil and cytidine triphosphate (CTP). A prerequisite for the brain’s utilization of cytidine is its conveyance from the circulation into the brain’s extracellular fluid and its incorporation into the neurons and glia. The effectual mechanism facilitating the brain’s uptake of cytidine has yet to be identified [22].

**Figure 4:**
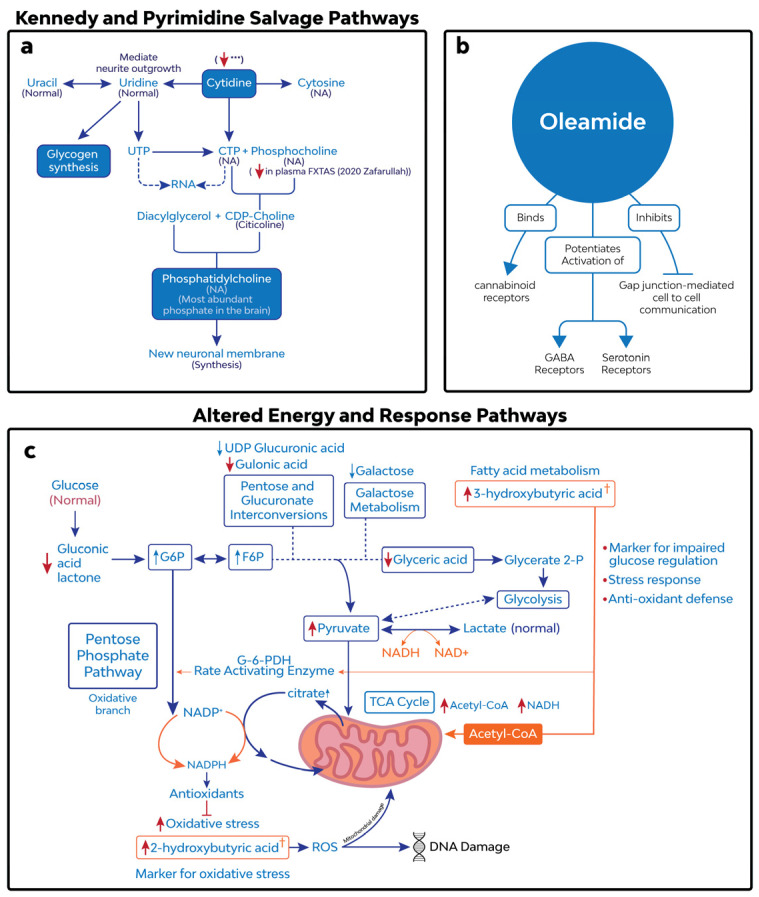
(**a**) Kennedy and pyrimidine salvage pathways, adapted from Cansev 2006 [22]. (**b**) Diagram showing interactions of oleamide in CNS. (**c**) Several metabolites involved in energy homeostasis and stress response pathways were identified to have changes in relative abundance in FXTAS. Red arrows show changes in the relative abundance of metabolites in the FXTAS group compared to controls. † Metabolites with discriminatory power between the FXTAS cases and controls.

Given the diminished relative abundance of cytidine in the brains affected by FXTAS, it is reasonable to anticipate dysregulations in the Kennedy and pyrimidine salvage pathways (Figure 4a). These pathways are involved in polysaccharide and phospholipid biosynthesis, detoxification processes, and protein and lipid glycosylation [23]. The disruption of new neuronal membrane synthesis could be linked to the etiology of neurodegeneration [24]. Interestingly, higher cytidine levels were observed in the biofluids of a small cohort of patients with various neurodegenerative dementias, including Alzheimer’s, frontotemporal dementia, and Lewy body dementia [25], and they have been postulated as a plasma marker for Alzheimer’s disease [26]. However, each form similarly involves the degradation of vulnerable neurons of various brain regions and shares a related symptomology to FXTAS, such as cognitive decline and impairments of body movement [27,28,29]. Changes in cytidine abundance were not reported in the plasma from FXTAS patients; however, a decreased level of choline phosphate, an intermediate of the Kennedy pathway, was observed in *FMR1* premutation carriers who had developed FXTAS [5]; a lower abundance of cytidine was also found in the cerebellum of premutation mice [6]. A previous open-label pilot clinical trial of citicoline, an intermediate in the Kennedy pathway, did not show clinical efficacy (expected as a 20% improvement on the FXTAS rating scale); however, after one year of treatment, none of the participants had significantly worse scores from the baseline [30].

Additional studies are necessary to understand the FXTAS-related biological abnormalities involved in cellular membrane synthesis, including those involved in the synthesis of phospholipids, which are essential for membrane fusion, mitochondrial stability, protein biosynthesis, and oxidative phosphorylation [31]. Further analysis of lipids in brains with FXTAS is needed to fully evaluate the impact of having a lower abundance of cytidine in the critical pathways of phospholipid biosynthesis and lipid glycosylation.

### 4.2. Brains with FXTAS Present with a High Abundance of Markers for Oxidative Stress

A higher relative abundance of 2-hydroxybutanoic acid (2HB) was observed within the ITG and CB in the FXTAS cases when compared to the controls. The abundance increased in the ITG of the FXTAS cases with increasing CGG repeat expansions (Supplemental Materials Figure S1). 2HB is formed from its derivative alpha-ketobutyrate (AKB), a by-product of threonine and methionine catabolism and glutathione (GSH) anabolism [32,33]. GSH is the primary intracellular antioxidant, and its production is increased during increased oxidative stress [34]. Under normal conditions, AKB is converted to propionyl-CoA, a key player in the citric acid cycle (TCA). When AKB production outpaces its catabolism, 2HB is formed. A high NADH/NAD+ ratio potentiates this process by increasing the activity of lactate dehydrogenase, an enzyme that catalyzes the conversion of AKB to 2HB [33] (Figure 4c).

In addition, an increased 2HB level is a well-established biomarker for insulin resistance (IR), impaired glucose tolerance, metabolic acidosis, and oxidative stress [33,34,35,36]. The aforementioned NADH/NAD+ imbalance may be attributed to increased lipid oxidation, a common feature of IR and a source of oxidative stress. Increased 2HB concentrations have also been reported in biofluids in schizophrenia patients [37], inherited metabolic diseases [38], and multiple sclerosis [39], and it showed discriminatory power between the FXTAS cases and controls in our analysis (Figure 3f,m). Interestingly, Giulivi et al. reported a lower 2HB plasma abundance in premutation carriers compared to controls [4]. However, a second study reported an increased abundance of 2HB in plasma collected from a larger cohort of *FMR1* premutation carriers compared to healthy controls. The same study did not find a difference in the relative abundance of 2HB between premutation carriers that had developed FXTAS and those that had not during the study period [5] (Table 4). To date, no study has correlated abnormal 2HB concentrations to specific FXTAS symptoms or other neurodegenerative conditions.

The relative abundance of proline was decreased in the ITG and CB of the FXTAS cases. Contrarily, the abundance of proline was increased in the plasma of *FMR1* premutation carriers (Table 4) [4,21]. Prior studies on the regulatory roles of proline biosynthesis suggested the role of the proline cycle in augmenting redox cycling for the Warburg effect, increasing the level of total pyridine nucleotides and increasing the capacity for antioxidant defenses by stimulating the oxidative branch of the pentose phosphate pathway [40].

Further, the relative abundance of the nucleoside xanthosine was increased (adj. *p*-value 0.061) in the CB of the FXTAS cases with a clinical diagnosis of dementia. Purine metabolism is a component of the mitochondrial response to oxidative stress and may be involved in slowing progressive mitochondrial dysfunction. High levels of xanthosine have been reported in the plasma of patients with untreated schizophrenia [41]. Changes in xanthosine and guanine expression were also detected in postmortem entorhinal cortex in AD [42].

### 4.3. Dysfunction in Energy Metabolism in Brains with FXTAS

Our analysis found a trending (adj. *p*-value 0.077) increased relative abundance of 3-hydroxybutiric acid, a fatty acid and marker for impaired glucose regulation, and 1,5-anhydroglucitol, a glucose analog in ITG, as well as a significantly (adj. *p*-value 0.0018) decreased relative abundance of oleamide in the CB. The volcano plots additionally identified fructose-1-phosphate and glucose-6-phosphate to be differentially expressed in the CB, while intermediate metabolites involved in glycolysis, including glyceric acid and pyruvic acid, were differentially expressed in the ITG (Figure 1a,b), demonstrating a dysfunction in glucose utilization (Figure 4c). These results provide additional evidence supporting the presence of altered energy metabolism in the brains of FXTAS individuals. Fatty acids are among the most important lipid molecules that determine the brain’s integrity and function [43]. Changes in the plasma metabolic profile of fatty acids have been reported in patients diagnosed with FXTAS [5] and in various neurodegenerative and neuropsychiatric disorders such as Alzheimer’s disease, autism spectrum disorders, schizophrenia, anxiety, and FXTAS [44,45,46].

### 4.4. A Central-Acting Fatty Acid Shows Lower Abundance in FXTAS

The relative abundance of oleamide was significantly lower (logFC: −1.06, adj. *p*-value 0.0018) in the CB with FXTAS (Figure 2e). In addition, oleamide showed an excellent discriminatory power during the ROC analysis (Figure 3h). Oleamide is a fatty acid primary amide, which exerts effects on vascular and neuronal tissues. The recognized biological effects of oleamide include the binding of cannabinoid receptors [47], specifically CB1 [48,49], immunological suppression, sleep induction, serotonin and gamma-aminobutyric acid receptor activation [50,51], and the inhibition of gap junction [52] coupling (Figure 4b). In addition, in a mouse model of AD, exogenous oleamide supplementation reduced amyloid-β (Aβ) accumulation and suppressed inflammation after amyloid Aβ deposition by enhancing microglial phagocytosis [53]. Furthermore, oleamide may elicit protective effects against excitotoxicity-induced neuronal death, partly related to its direct and/or indirect calpain inhibitory activity [54] and other yet unknown biological actions.

Gap junctions are found between neurons and glial cells [52]. Gap junctions play an important role in neuronal activity and are implicated in many cognitive processes, including the modulation of memory processes, brain development, and the mechanisms of psychostimulant dependence [55]. In addition, glial gap junctions modulate the expression of hundreds of genes [56] and enable the propagation of signaling molecules implicated in cerebrovascular regulation [57] and astrocytic glutamate release [58,59,60,61]. A low relative abundance of oleamide in brains with FXTAS may therefore affect neuronal excitability and facilitate a higher permeability by microvascular endothelial cells. A lack of the beneficial effects of oleamide in brains with FXTAS may also contribute to lowering the brain’s capacity to respond to the damage caused by inflammation and excitotoxicity, as well as promoting a higher accumulation of Aβ in brains with FXTAS and concomitant AD. Decreased oleamide plasma levels were reported in *FMR1* premutation carriers and proposed as a potential biomarker for the diagnosis of FXTAS [21], showing a negative correlation with CGG expansion [4], which was also identified in our analysis. A lower abundance of oleamide in plasma has also been postulated as a marker for AD [62].

### 4.5. Study Advantages

Submitting a variety of tissues to metabolomic studies is important as each tissue has its own unique advantages. For example, CSF is less prone to metabolite degradation in comparison to plasma because of its relatively decreased enzymatic activity [63]. Plasma, however, is easier to obtain in larger quantities. In the study of neurogenerative disease, metabolomic analysis of brain tissue offers the advantage of the direct quantification of metabolites at the site of the organ of interest. This helps limit the confounding variables introduced by the analysis of tissues separate from the brain. This is especially relevant in the case of brains where the blood–brain barrier (BBB) and the blood–CSF barrier (BSFB) reduce the influx and efflux of small molecules such as metabolites [64].

Using a combination of tissues helps to strengthen the search for disease biomarkers. Metabolites found in multiple tissues to be abnormally altered compared to controls raises confidence in results being related to a disease rather than an artifact of pretreatment, sampling, or some other unknown variable impacting a single tissue type. In addition, finding biomarkers of disease to be universal across a tissue will allow for easier disease staging via the testing of more readily acquired tissue, such as plasma. We compared our results to previously reported metabolomics analyses conducted in plasma from *FMR1* premutation with and without FXTAS patients and frozen brain tissue from a mouse model of FXTAS (Table 4).

### 4.6. Study Limitations

Differences in the postmortem interval (PMI) pose a significant limitation. Certain changes in metabolite concentrations might not necessarily indicate disease pathology, but rather a difference between antemortem and postmortem conditions. Several studies have found metabolites altered by PMI in a variety of tissues [65,66,67]. Studies in sheep and human brains found free trimethylammonium (fTMA), propionate, and butyrate to be elevated as PMI increased [68,69]. Our analysis was limited to primary metabolites using GC-MS quantification. Further analysis of lipid metabolomics is granted to validate our findings and for a more comprehensive comparison between lipid metabolic disturbances found in the plasma of *FMR1* premutation carriers and FXTAS patients.

## 5. Conclusions

An altered abundance of some metabolites may influence the clinical outcome of patients with FXTAS. Our data identified alterations in the metabolic pathways related to oxidative stress responses and bioenergetics in the human postmortem brains with FXTAS. The results from our study demonstrate similarities with previous metabolic disturbances reported in plasma of *FMR1* premutation carriers and FXTAS patients, but they also demonstrate differences that need further analysis. The significantly lower abundance of cytidine and oleamide in the FXTAS brains and their discriminatory potential between diseased and healthy brain tissue should serve as starting point to study their potential protective effects against neuronal damage in FXTAS. Oleamide and other metabolites of interest previously reported in plasma and now reported in postmortem brains suggest their potential as biomarkers for the diagnosis and prognosis of FXTAS.

## Figures and Tables

**Table 1:** Subject characteristics.

		Control (*n* = 19)	Case (*n* = 25)	*p*-Value
Age				0.868
	N	19	25	
	Mean (SD)	80.5 (8.5)	80.8 (7.2)	
	Median (Range)	80 (62–97)	81 (67–93)	
PMI				0.939
	N	19	18	
	Mean (SD)	23.5 (33)	21.5 (28.7)	
	Median (Range)	8.7 (2.3–136.1)	15.2 (2.5–127)	
Gender				1
	F	9 (47.4%)	12 (48%)	
	M	10 (52.6%)	13 (52%)	
Blood CGG				
	N	19	25	
	Mean (SD)	30.6 (4.8)	82 (15.7)	
	Median (Range)	30 (23–42)	81 (55–120)	
	20–44	19 (100%)	0	
	45–54	0	0	
	55–74	0	7 (28%)	
	75–94	0	14 (56%)	
	95–120	0	4 (16%)	
Race/Ethnicity				
	White	19 (100%)	25 (100%)	

**Table 2:** Differential abundance cases/controls.

Inferior Temporal Gyrus						Cerebellum					
BinBase Name	Biological Role	PubChem	logFC	*p*-Value	adj. *p*-Value	BinBase Name	Biological Role	PubChem	logFC	*p*-Value	adj. *p*-Value
Cytidine	nucleoside	6175	−1.381	**0.000004**	**0.0007**	Oleamide	fatty acid amide	5283387	−1.060	**0.00001**	**0.0018**
3-hydroxybutyric acid	ketone body	92135	1.075	0.0011	0.0772	Fructose-1-phosphate	monosaccharide	439394	1.362	0.0013	0.1067
2-hydroxybutanoic acid	organic acid	440864	0.939	0.0015	0.0772	1,5-anhydroglucitol	monosaccharide	64960	1.037	0.0039	0.1683
1,5-anhydroglucitol	monosaccharide	64960	1.083	0.0016	0.0772	Phosphoethanolamine	phosphomonoester	1015	0.569	0.0046	0.1683
Oleamide	fatty acid amide	5283387	−0.904	0.0029	0.1115	2-hydroxybutanoic acid	organic acid	440864	1.056	0.005	0.1683
Guanine	amino acid/purine	764	0.747	0.0043	0.1181	*N*-acetylmannosamine	amino sugar	11096158	−0.779	0.0069	0.1796
Xanthine	amino acid/purine	1188	−0.347	0.0043	0.1181	Lysine	essential amino acid	5962	−0.398	0.0074	0.1796
Histidine	essential amino acid	6274	-0.624	0.0057	0.138	Maltotriose	trisaccharide	439586	0.908	0.0103	0.2134
Proline	essential amino acid	145742	−0.730	0.0094	0.1854	Proline	essential amino acid	145742	−0.909	0.0114	0.2134
Phosphoric acid	organic acid	1004	0.487	0.0096	0.1854	Threonine	essential amino acid	6288	−0.323	0.0141	0.2353
Gluconic acid	organic acid	6857417	−0.952	0.0118	0.207	Maltose	disaccharide	439186	0.955	0.0161	0.2353
Cysteine	amino acid	5862	−0.814	0.0139	0.2232	Xylose	monosaccharide	135191	0.658	0.0167	0.2353
Cholesterol	lipid	5997	0.354	0.0168	0.2493	Erythritol	monosaccharide polyol	222285	0.434	0.0234	0.2906
Threonine	essential amino acid	6288	−0.265	0.0181	0.2493	Glycyl tyrosine	dipeptide	92829	−0.591	0.0241	0.2906
Maltotriose	trisaccharide	439586	0.752	0.0199	0.2557	Inosine	nucleoside	6021	0.780	0.0268	0.3021
Glyceric acid	organic acid	752	−1.710	0.0219	0.2592	Ascorbic acid	organic acid	54670067	0.480	0.0294	0.3107
Pyrophosphoric acid	inorganic acid	1023	0.446	0.0228	0.2592	Glycerol	sugar alcohol	753	−0.349	0.0319	0.315
Pyruvic acid	organic acid	1060	1.019	0.03	0.3145	Histidine	essential amino acid	6274	−0.602	0.0336	0.315
Erythrose	monosaccharide	439574	−0.461	0.031	0.3145	Arabitol	sugar alcohol	94154	0.364	0.0356	0.3162
Adipic acid	organic acid	196	0.283	0.0405	0.3904	Serine	non-essential amino acid	5951	−0.342	0.0413	0.3466
Gluconic acid lactone	organic acid	7027	−0.653	0.0451	0.4145	Leucine	essential amino acid	6106	−0.329	0.0486	0.3466
Adenosine	nucleoside	60961	0.639	0.0474	0.4161						

**Table 3:** Enriched pathways in FXTAS vs. controls.

ITG						
Pathway Name	BinBase Name	logFC	*p*-Value	adj. *p*-Value	t	KEGG
	Histidine	−0.624	**0.006**	0.138	−2.912	C00135
Protein digestion and absorption Homo sapiens (human) hsa04974 *p*-value: **0.0019** adj. *p*-value **0.009** q-value **0.0039** Normalized enrichment score: **−1.948**	Proline	−0.730	**0.009**	0.185	−2.723	C00148
	Cysteine	−0.814	**0.014**	0.223	−2.568	C00097
	Threonine	−0.265	**0.018**	0.249	−2.460	C00188
	Methionine	−0.284	0.091	0.446	−1.728	C00073
	Leucine	−0.194	0.213	0.585	−1.265	C00123
	Glutamine	0.187	0.442	0.737	0.776	C00064
	Aspartic acid	−0.085	0.608	0.803	−0.516	C00049
	Alanine	−0.066	0.708	0.870	−0.377	C00041
	Aryptophan	−0.038	0.799	0.891	−0.256	C00078
Aminoacyl tRNA biosynthesis Homo sapiens (human) hsa00970 *p*-value: **0.0053** adj. *p*-value **0.0132** q-value **0.0056** Normalized enrichment score: **−1.868**	Histidine	−0.624	**0.006**	0.138	−2.912	C00135
	Threonine	−0.265	**0.018**	0.249	−2.460	C00188
	Isoleucine	−0.276	0.059	0.446	−1.938	C00407
	Glycine	−0.236	0.065	0.446	−1.896	C00037
	Serine	−0.252	0.080	0.446	−1.816	C00065
	Asparagine	−0.139	0.273	0.643	−1.110	C00152
	O-phosphoserine	0.627	0.295	0.643	1.148	C01005
	Tyrosine	−0.102	0.547	0.801	−0.608	C00082
	Lysine	−0.074	0.622	0.811	−0.497	C00047
	Tryptophan	−0.038	0.799	0.891	−0.256	C00078
D-amino acid metabolism Homo sapiens (human) hsa00470 *p*-value: **0.0391** adj. *p*-value **0.065** q-value **0.0274** Normalized enrichment score: **−1.641**	Proline	−0.730	**0.009**	0.185	−2.723	C00148
	Cysteine	−0.814	**0.014**	0.223	−2.568	C00097
	Threonine	−0.265	**0.018**	0.249	−2.460	C00188
	Methionine	−0.284	0.091	0.446	−1.728	C00073
	Glutamic acid	−0.123	0.262	0.629	−1.137	C00025
	*N*-acetylglutamate	−0.171	0.321	0.671	−1.006	C00624
	Trans-4-hydroxyproline	0.139	0.350	0.675	0.946	C01157
	Glutamine	0.187	0.442	0.737	0.776	C00064
	Aspartic acid	−0.085	0.608	0.804	−0.516	C00049
	Alanine	−0.066	0.708	0.870	−0.377	C00041
	Phenylalanine	−0.007	0.964	0.969	−0.045	C00079
**CB**						
**Pathway Name**	**Pathway Name**	**logFC**	***p*-Value**	**Adjusted *p*-Value**	**t**	**KEGG**
Protein digestion and absorption Homo sapiens (human) hsa04974 *p*-value: **0.0004** adj. *p*-value **0.002** q-value **0.0009** Normalized enrichment score: **−2.017**	Proline	−0.909	**0.011**	0.213	−2.649	C00148
	Threonine	−0.322	**0.014**	0.235	−2.562	C00188
	Histidine	−0.602	**0.034**	0.315	−2.198	C00135
	Leucine	−0.329	**0.049**	0.347	−2.031	C00123
	Cysteine	−0.618	0.115	0.448	−1.609	C00097
	Methionine	−0.313	0.124	0.448	−1.570	C00073
	Alanine	−0.330	0.137	0.448	−1.518	C00041
	Aspartic acid	−0.272	0.155	0.469	−1.447	C00049
	Glutamine	0.148	0.531	0.806	0.632	C00064
	Tryptophan	−0.089	0.608	0.853	−0.517	C00078
D-amino acid metabolism Homo sapiens (human) hsa00470 *p*-value: **0.0034** adj. *p*-value **0.0069** q-value **0.0036** Normalized enrichment score: **−1.815**	Proline	−0.909	**0.011**	0.18	−2.814	C00148
	Threonine	−0.322	**0.014**	0.235	−2.562	C00188
	Cysteine	−0.618	0.115	0.315	−2.198	C00097
	Methionine	−0.313	0.124	0.347	−2.112	C00073
	Alanine	−0.33	0.137	0.347	−2.018	C00041
	Aspartic acid	−0.272	0.155	0.473	−1.427	C00049
	Phenylalanine	−0.126	0.420	0.731	−0.815	C00079
	Glutamine	0.148	0.531	0.806	0.632	C00064
	Trans-4-hydroxyproline	−0.073	0.702	0.895	−0.386	C00157
	*N*-acetylglutamate	−0.022	0.902	0.982	−0.124	C00624
	Glutamic acid	−0.008	0.954	0.982	−0.058	C00025

**Table 4:** Metabolomics in *FMR1* premutation.

ITG				
BinBase Name	*FMR1* FXTAS Human Frozen Brain	Zafarulla 2020 [5] *FMR1* FXTAS Human Plasma	Giulivi 2016 [4,21] *FMR1* Premutation Human Plasma	
3-hydroxybutyric acid	↑	↑	↓	
2-hydroxybutanoic acid	↑	↑	↓	
Oleamide	↓		↓	
Histidine	↓		↓	
Proline	↓		↑	
Gluconic acid	↓		↑	
Glyceric acid	↓		↑	
Pyruvic acid	↑	↑		
**CB**				
**BinBase name**	***FMR1* FXTAS** **Human** **Frozen brain**	**Zafarulla 2020 [5]** ***FMR1* FXTAS** **Human Plasma**	**Giulivi 2016 [4,21]** ***FMR1* premutation** **Human Plasma**	**Kong [6]** ***FMR1* mouse** **Frozen CB**
Oleamide	↓		↓	
1,5-anhydroglucitol	↑			No change
Phosphoethanolamine	↑	↑		No change
2-hydroxybutanoic acid	↑	↑	↓	
Lysine	↓		↓	↑
Proline	↓		↑	No change
Threonine	↓			No change
Maltose	↑		↑	
Xylose	↑		↑	
Erythritol	↑			No change
Inosine	↑			↑
Glycerol	↓	↑	↑	↓
Histidine	↓		↓	No change
Serine	↓			No change
Leucine	↓			↑
↑ Increased, ↓ decreased				
	Similar results between FXTAS frozen brain and plasma from premutation carriers with FXTAS			
	Similar results between FXTAS frozen brain and plasma from premutation carriers without FXTAS			
	Similar results between frozen cerebellum in FXTAS cases and an FMR1 mouse model			

## Data Availability

All metabolomics data generated, in both raw and processed format, are available at the Metabolomics Workbench www.metabolomicsworkbench.org (accessed on 22 August 2023).

## Supplementary Materials

The following supporting information can be downloaded at: https://www.mdpi.com/article/10.3390/cells12172132/s1, Table S1: List of identified metabolites in ITG; Table S2: List of identified metabolites in CB; Table S3: Clinical and pathological measures; Figure S1: Correlation of metabolite abundance with CGG expansion.
