# Supplementary material for: Brain Metabolomics in Fragile X-Associated Tremor/Ataxia Syndrome (FXTAS)
Source: Cells. 2023 Aug 23;12(17):2132. doi: 10.3390/cells12172132 (PMC10487256; doi:10.3390/cells12172132)
Supplement: Supplementary file 1 [file cells-12-02132-s001.zip › cells-2446161-supplementary.pdf]

Article

Brain Metabolomics in Fragile X-Associated Tremor/Ataxia Syndrome (FXTAS)

Maria Jimena Salcedo-Arellano [1,2,3], Michael D. Johnson [1,3], Yingratana A. McLennan [1,3], Ye Hyun Hwang [4], Pablo Juarez [1,3], Erin Lucille McBride [1,3], Adriana P. Pantoja [1,3], Blythe Durbin-Johnson [5], Flora Tassone [2,4], Randi J. Hagerman [2,6] and Verónica Martínez-Cerdeño [1,2,3,*]

1. Department of Pathology and Laboratory Medicine, UC Davis School of Medicine, Sacramento, CA 95817, USA; mjsalcedo@ucdavis.edu (M.J.S.-A.); mdajohnson@ucdavis.edu (M.D.J.); yamclennan@ucdavis.edu (Y.A.M.); pjuarez@ucdavis.edu (P.J.); elmcb@ucdavis.edu (E.L.M.); adpantoja@ucdavis.edu (A.P.P.); vmartinezcerdeno@ucdavis.edu (V.M.-C.)
2. Medical Investigation of Neurodevelopmental Disorders (MIND) Institute, University of California Davis, Sacramento, CA 95616, USA; mjsalcedo@ucdavis.edu (M.J.S.-A.); ftassone@ucdavis.edu (F.T.); randijhagerman@ucdavis.edu (R.J.H.); vmartinezcerdeno@ucdavis.edu (V.M.-C.)
3. Institute for Pediatric Regenerative Medicine at Shriners Hospitals for Children Northern California, Sacramento, CA 95817, USA; mjsalcedo@ucdavis.edu (M.J.S.-A.); mdajohnson@ucdavis.edu (M.D.J.); yamclennan@ucdavis.edu (Y.A.M.); pjuarez@ucdavis.edu (P.J.); elmcb@ucdavis.edu (E.L.M.); adpantoja@ucdavis.edu (A.P.P.); vmartinezcerdeno@ucdavis.edu (V.M.-C.)
4. Department of Biochemistry and Molecular Medicine, UC Davis School of Medicine, Sacramento, CA 95817, USA; yehhwang@ucdavis.edu (Y.H.H.); ftassone@ucdavis.edu (F.T.)
5. Division of Biostatistics, Department of Public Health Sciences, UC Davis School of Medicine, Sacramento, CA 95817, USA; bpdurbin@ucdavis.edu
6. Department of Pediatrics, UC Davis School of Medicine, Sacramento, CA 95817, USA; randijhagerman@ucdavis.edu

* Correspondence: vmartinezcerdeno@ucdavis.edu

Author Contributions: Conceptualization, M.J.S.-A. and V.M.-C.; methodology, M.J.S.-A. and V.M.-C.; formal analysis, M.J.S.-A., B.D.-J. and V.M.-C.; investigation, M.J.S.-A., M.D.J., Y.A.M., E.L.M., A.P.P., P.J. and Y.H.H.; data curation, West Coast Metabolomics Center; writing—original draft preparation, M.J.S.-A., M.D.J., Y.A.M., E.L.M., A.P.P., P.J. and Y.H.H.; writing—review and editing, B.D.-J., F.T., R.J.H. and V.M.-C.; visualization, M.J.S.-A. and M.D.J.; supervision, V.M.-C.; project administration, M.J.S.-A. and V.M.-C.; funding acquisition, V.M.-C. and R.J.H. All authors have read and agreed to the published version of the manuscript.

Supplementary Material

Table S1 List of metabolites identified in ITG

BinBase name	PubChem	logFC	P.Value	adj.P.Val	t	KEGG
**1-monopalmitin**	14900	0.119	0.525	0.800	0.643	NA
**1-monostearin**	24699	0.326	0.208	0.585	1.279	D01947
**1,2-anhydro-myo-inositol**	119054	-0.248	0.433	0.732	-0.794	NA
**1,5-anhydroglucitol**	64960	1.083	**0.002**	**0.077**	3.374	C07326
**2-aminobutyric acid**	6657	0.295	0.264	0.629	1.132	C02721
**2-deoxypentitol**	249377	0.283	0.337	0.675	0.973	NA
**2-deoxytetronic acid**	192742	0.145	0.656	0.833	0.449	NA
**2-hydroxybutanoic acid**	440864	0.939	**0.002**	**0.077**	3.387	C05984
**2-hydroxyglutaric acid**	43	0.316	0.100	0.446	1.680	C02630
**2-hydroxyvaleric acid**	98009	0.152	0.500	0.790	0.682	NA
**2-monoolein**	5319879	-0.100	0.732	0.877	-0.345	NA
**2,5-dihydroxypyrazine**	23368901	0.423	0.245	0.620	1.178	NA
**3-aminoisobutyric acid**	64956	-0.163	0.730	0.877	-0.356	C05145
**3-hydroxy-3-methylglutaric acid**	1662	-0.321	0.159	0.519	-1.435	C03761
**3-hydroxybutyric acid**	92135	1.074	**0.001**	**0.077**	3.511	C01089
**3-phosphoglycerate**	724	-0.522	0.104	0.446	-1.662	C00597
**4-aminobutyric acid**	119	0.589	0.066	0.446	1.888	C00334
**4-hydroxybutyric acid**	10413	0.387	0.088	0.446	1.759	C00989
**4-hydroxyphenylacetic acid**	127	0.177	0.654	0.833	0.452	C00642
**5-aminovaleric acid**	138	-0.568	0.215	0.585	-1.385	C00431
**5-methoxytryptamine**	1833	0.025	0.952	0.969	0.063	C05659
**5'-deoxy-5'-methylthioadenosine**	439176	0.039	0.868	0.921	0.167	C00170
**acetaminophen**	1983	0.091	0.920	0.960	0.105	C06804
**adenine**	190	0.364	0.101	0.446	1.679	C00147
**adenosine**	60961	0.639	0.047	0.416	2.042	C00212
**adenosine-5-monophosphate**	6083	0.401	0.124	0.462	1.568	C00020
**adipic acid**	196	0.283	0.040	0.390	2.130	C06104
**alanine**	5950	-0.066	0.708	0.870	-0.377	C00041
**alloxanoic acid**	94146	0.233	0.591	0.801	0.542	NA
**allylbenzene**	9309	-0.180	0.731	0.877	-0.360	NA
**alpha-aminoadipic acid**	92136	0.088	0.761	0.887	0.307	C00956
**aminomalonate**	100714	-0.758	0.084	0.446	-1.943	C00872
**arabitol**	94154	0.253	0.151	0.510	1.464	C01904
**arachidic acid**	10467	-0.112	0.494	0.788	-0.690	C06425
**arachidonic acid**	444899	0.069	0.580	0.801	0.558	C00219
**ascorbic acid**	54670067	0.207	0.291	0.643	1.070	C00072
**asparagine**	6267	-0.139	0.273	0.643	-1.110	C00152
**aspartic acid**	5960	-0.085	0.608	0.804	-0.516	C00049
**azelaic acid**	19347555	0.189	0.505	0.792	0.673	C08261
**behenic acid**	8215	0.177	0.385	0.704	0.880	C08281

**beta-alanine**	239	0.044	0.782	0.887	0.278	C00099
**beta-alanyl lysine**	440638	-0.144	0.591	0.801	-0.543	C05341
**beta-glycerolphosphate**	2526	0.325	0.170	0.539	1.395	C02979
**beta-sitosterol**	222284	-0.419	0.402	0.705	-0.901	C01753
**butyrolactam**	12025	-0.214	0.457	0.741	-0.751	NA
**caproic acid**	8892	0.006	0.981	0.981	0.024	C01585
**cholesterol**	5997	0.354	0.017	0.249	2.491	C00187
**cholesterone mino**	91477	0.054	0.804	0.892	0.250	C00599
**cis-gondoic acid**	5282768	0.239	0.343	0.675	0.960	C16526
**citric acid**	311	0.215	0.391	0.704	0.868	C00158
**citrulline**	9750	-0.352	0.154	0.513	-1.451	C00327
**conduritol-beta-epoxide**	9989541	0.253	0.565	0.801	0.581	NA
**creatinine**	588	0.190	0.051	0.424	2.013	C00791
**cystathionine**	439258	0.133	0.777	0.887	0.286	C02291
**cysteine**	5862	-0.814	0.014	0.223	-2.568	C00097
**cysteine sulfonic acid MassBank**	129636156	-0.329	0.294	0.643	-1.067	NA
**cysteine-glycine**	439498	-0.094	0.810	0.893	-0.243	C01419
**cystine**	595	-0.564	0.108	0.449	-1.645	C01420
**cytidine**	6175	-1.381	**0.000**	**0.001**	-5.506	NA
**D-erythro-sphingosine**	5280335	0.151	0.527	0.800	0.638	C00319
**dehydroabietic acid**	94391	-0.267	0.612	0.804	-0.535	C12078
**dehydroascorbic acid**	440667	0.572	0.088	0.446	1.747	C05422
**diethanolamine**	8113	-0.673	0.260	0.629	-1.146	C06772
**dihydrocholesterol**	66066	0.413	0.137	0.499	1.520	C12978
**dihydroxyacetone**	670	0.063	0.888	0.932	0.141	C00184
**erythritol**	222285	-0.209	0.405	0.705	-0.841	C00503
**erythronic acid lactone isomer**	5325915	0.175	0.296	0.643	1.058	C02341
**erythrose**	439574	-0.461	0.031	0.315	-2.243	C01796
**ethanolamine**	700	-0.318	0.053	0.424	-1.993	C00189
**fructose**	439709	0.394	0.250	0.620	1.167	C02336
**fructose-1-phosphate**	439394	0.428	0.411	0.708	0.833	C01094
**fructose-6-phosphate**	440641	0.566	0.301	0.645	1.049	C05345
**fucose**	439650	-0.241	0.087	0.446	-1.750	C02095
**fumaric acid**	444972	0.194	0.353	0.675	0.939	C00122
**galactinol**	11727586	0.340	0.121	0.460	1.588	NA
**galactose**	439357	-0.712	0.667	0.842	-0.444	C00984
**gluconic acid**	6857417	-0.952	0.012	0.207	-2.633	C00800
**gluconic acid lactone**	7027	-0.653	0.045	0.414	-2.065	C00198
**glucose**	64689	0.183	0.770	0.887	0.295	C00221
**glucose-1-phosphate**	65533	0.165	0.360	0.682	0.925	C00103

**glucose-6-phosphate**	5958	0.681	0.402	0.705	0.847	C00092
**glutamic acid**	33032	-0.123	0.262	0.629	-1.137	C00025
**glutamine**	5961	0.187	0.442	0.737	0.776	C00064
**glutaric acid**	743	0.132	0.575	0.801	0.566	C00489
**glyceric acid**	752	-1.710	0.022	0.259	-2.766	C00258
**glycerol**	753	-0.152	0.289	0.643	-1.073	C00116
**glycerol-3-galactoside**	16048618	0.263	0.227	0.593	1.226	C05401
**glycerol-alpha-phosphate**	754	0.386	0.170	0.539	1.397	C03189
**glycine**	750	-0.236	0.065	0.446	-1.896	C00037
**glycolic acid**	757	0.063	0.656	0.833	0.448	C00160
**glycyl tyrosine**	92829	-0.139	0.592	0.801	-0.541	NA
**glycyl-glycine**	11163	0.050	0.830	0.901	0.217	C02037
**glycyl-proline**	3013625	-0.342	0.204	0.585	-1.295	NA
**guanine**	764	0.747	0.004	0.118	3.044	C00242
**guanosine**	6802	0.292	0.236	0.608	1.202	C00387
**heptadecanoic acid**	10465	-0.047	0.784	0.887	-0.276	NA
**hexadecylglycerol NIST**	72733	-0.281	0.594	0.801	-0.564	C13859
**histidine**	6274	-0.624	0.006	0.138	-2.912	C00135
**homocystine**	10010	-0.271	0.286	0.643	-1.085	C01817
**hypotaurine**	107812	0.212	0.452	0.740	0.760	C00519
**hypoxanthine**	790	-0.140	0.251	0.620	-1.165	C00262
**ile-ile**	7010568	0.200	0.447	0.737	0.770	C12H24N2O3
**inosine**	6021	0.361	0.206	0.585	1.285	C00294
**inositol-4-monophosphate**	440043	0.077	0.769	0.887	0.296	C03546
**isoleucine**	6306	-0.276	0.059	0.446	-1.938	C00407
**isothreonic acid**	151152	0.052	0.722	0.877	0.358	C00639
**lactamide**	94220	-0.237	0.522	0.800	-0.666	NA
**lactic acid**	612	-0.014	0.959	0.969	-0.052	C01432
**lactose**	6134	0.258	0.423	0.723	0.809	C01970
**lactulose**	11333	-0.174	0.781	0.887	-0.291	C07064
**leucine**	6106	-0.194	0.213	0.585	-1.265	C00123
**levoglucosan**	2724705	-0.367	0.221	0.591	-1.245	C22350
**linoleic acid**	5280450	-0.020	0.928	0.963	-0.091	C01595
**lysine**	5962	-0.074	0.622	0.811	-0.497	C00047
**maleimide**	10935	-0.802	0.214	0.585	-1.338	C07272
**malic acid**	525	0.086	0.696	0.861	0.393	C00711
**maltose**	439186	0.647	0.063	0.446	1.911	C00208
**maltotriose**	439586	0.752	0.020	0.256	2.444	C01835
**mannitol**	6251	0.037	0.947	0.969	0.068	C00392

**mannose**	18950	-0.159	0.786	0.887	-0.273	C00159
**methanolphosphate**	13130	0.300	0.115	0.454	1.609	NA
**methionine**	6137	-0.284	0.091	0.446	-1.728	C00073
**methionine sulfoxide**	158980	-0.041	0.836	0.901	-0.209	C02989
**myo-inositol**	892	0.254	0.098	0.446	1.697	C00137
**myristic acid**	11005	-0.248	0.560	0.801	-0.616	C06424
**N-acetyl-D-galactosamine**	440552	-0.270	0.196	0.585	-1.320	C05021
**N-acetylaspartate**	65065	0.179	0.389	0.704	0.871	C01042
**N-acetylglutamate**	70914	-0.171	0.321	0.671	-1.006	C00624
**N-acetylmannosamine**	11096158	-0.054	0.820	0.900	-0.229	NA
**N-acetylmethionine MassBank**	448580	-0.230	0.113	0.454	-1.627	C02712
**N-acetylneuraminate MassBank**	439197	-0.217	0.175	0.546	-1.384	C00270
**N-carbamylglutamate**	121396	-0.361	0.145	0.510	-1.484	C05829
**N-methyl-UMP**	44602426	0.267	0.337	0.675	0.975	NA
**nicotinamide**	936	-0.029	0.863	0.920	-0.173	C00153
**nicotinic acid MassBank**	938	0.175	0.150	0.510	1.470	C00253
**O-phosphoserine**	57689797	0.627	0.295	0.643	1.148	C01005
**octadecanol**	8221	0.624	0.121	0.460	1.589	NA
**octadecylglycerol**	3681	0.180	0.375	0.703	0.896	C13858
**oleamide**	5283387	-0.904	0.003	0.111	-3.180	C19670
**oleic acid**	445639	0.096	0.629	0.815	0.486	C00712
**ornithine**	6262	0.069	0.601	0.804	0.526	C00077
**oxoproline**	7405	0.056	0.539	0.800	0.619	C01879
**palmitic acid**	985	0.066	0.567	0.801	0.577	C00249
**palmitoleic acid**	445638	-0.128	0.551	0.801	-0.600	C08362
**pantothenic acid**	6613	-0.517	0.150	0.510	-1.467	C12276
**pentadecanoic acid**	13849	0.011	0.935	0.965	0.082	C16537
**phenylalanine**	6140	-0.007	0.964	0.969	-0.045	C00079
**phenylethylamine**	1001	-0.283	0.792	0.888	-0.276	C05332
**phosphate**	1004	0.487	0.010	0.185	2.714	C00009
**phosphoethanolamine**	1015	0.301	0.073	0.446	1.838	C00346
**proline**	145742	-0.730	0.009	0.185	-2.723	C00148
**propane-1,3-diol NIST**	10442	0.409	0.353	0.675	1.006	C02457
**pseudo uridine**	15047	0.173	0.521	0.800	0.647	C02067
**putrescine**	1045	0.362	0.210	0.585	1.274	C00138
**pyrophosphate**	1023	0.446	0.023	0.259	2.363	C00013
**pyruvic acid**	1060	1.019	0.030	0.315	2.574	C00022
**ribitol**	827	-0.130	0.387	0.704	-0.873	C00474
**ribonic acid**	5460677	-0.082	0.604	0.804	-0.522	C01685
**ribose**	5779	-0.208	0.109	0.449	-1.640	C00121

**salicylic acid**	338	0.419	0.077	0.446	1.821	C00805
**serine**	5951	-0.252	0.077	0.446	-1.816	C00065
**serotonin**	5202	-0.201	0.307	0.651	-1.034	C00780
**shikimic acid**	8742	0.067	0.843	0.904	0.200	C00493
**sorbitol**	5780	0.149	0.536	0.800	0.624	C00794
**spermidine**	1102	-0.171	0.582	0.801	-0.555	C00315
**squalene**	638072	-0.097	0.831	0.901	-0.222	C00751
**stearic acid**	5281	0.025	0.954	0.969	0.061	C01530
**succinate semialdehyde**	9543238	0.111	0.682	0.855	0.413	NA
**succinic acid**	1110	-0.180	0.536	0.800	-0.623	C00042
**sucrose**	5988	0.716	0.074	0.446	1.838	C00089
**terephthalic acid**	7489	0.172	0.227	0.593	1.229	C06337
**threitol**	169019	-0.334	0.190	0.582	-1.332	C16884
**threonic acid**	5460407	0.214	0.278	0.643	1.099	C01620
**threonine**	6288	-0.265	0.018	0.249	-2.460	C00188
**thymine**	1135	0.088	0.556	0.801	0.594	C00178
**tocopherol alpha-**	14985	0.066	0.755	0.887	0.315	C02477
**tocopherol gamma-**	92729	0.098	0.873	0.921	0.167	C02483
**trans-4-hydroxyproline**	5810	0.139	0.350	0.675	0.946	C01157
**tryptophan**	6305	-0.038	0.799	0.891	-0.256	C00078
**tyrosine**	6057	-0.102	0.547	0.801	-0.608	C00082
**UDP-glucuronic acid**	17473	-0.218	0.445	0.737	-0.773	C00167
**UDP-N-acetylglucosamine**	445675	0.053	0.692	0.861	0.400	C00043
**uracil**	1174	-0.183	0.468	0.752	-0.733	C00106
**urea**	1176	0.734	0.085	0.446	1.765	C00086
**uridine**	6029	-0.256	0.334	0.675	-0.978	C00299
**valine**	6287	-0.243	0.102	0.446	-1.671	C00183
**xanthine**	1188	-0.347	0.004	0.118	-3.020	C00385
**xanthosine**	64959	-0.054	0.743	0.885	-0.331	C01762
**xylitol**	6912	-0.348	0.101	0.446	-1.676	C00379
**xylose**	135191	-0.219	0.324	0.671	-0.999	C00181
**xylulose**	439205	-0.206	0.340	0.675	-0.966	C00312

Table S2 List of metabolites identified in CB

BinBase name	PubChem	logFC	P.Value	adj.P.Val	t	KEGG
**oleamide**	5283387	-1.060	**0.000**	**0.002**	-5.060	C19670
**fructose-1-phosphate**	439394	1.362	**0.001**	**0.107**	3.480	C01094
**1,5-anhydroglucitol**	64960	1.037	0.004	0.168	3.060	C07326
**phosphoethanolamine**	1015	0.569	0.005	0.168	2.992	C00346
**2-hydroxybutanoic acid**	440864	1.056	0.005	0.168	2.966	C05984
**N-acetylmannosamine**	11096158	-0.779	0.007	0.180	-2.853	NA
**lysine**	5962	-0.398	0.007	0.180	-2.814	C00047
**maltotriose**	439586	0.908	0.010	0.213	2.698	C01835
**proline**	145742	-0.909	0.011	0.213	-2.649	C00148
**threonine**	6288	-0.322	0.014	0.235	-2.562	C00188
**maltose**	439186	0.955	0.016	0.235	2.509	C00208
**xylose**	135191	0.658	0.017	0.235	2.494	C00181
**erythritol**	222285	0.434	0.023	0.291	2.353	C00503
**glycyl tyrosine**	92829	-0.591	0.024	0.291	-2.349	NA
**inosine**	6021	0.780	0.027	0.302	2.296	C00294
**ascorbic acid**	54670067	0.480	0.029	0.311	2.256	C00072
**glycerol**	753	-0.348	0.032	0.315	-2.221	C00116
**histidine**	6274	-0.602	0.034	0.315	-2.198	C00135
**arabitol**	94154	0.364	0.036	0.316	2.173	C01904
**serine**	5951	-0.342	0.041	0.347	-2.112	C00065
**leucine**	6106	-0.329	0.049	0.347	-2.031	C00123
**isoleucine**	6306	-0.297	0.050	0.347	-2.018	C00407
**dehydroascorbic acid**	440667	0.775	0.050	0.347	2.018	C05422
**N-acetylmethionine**	448580	-0.374	0.054	0.347	-1.987	C02712
**MassBank 2-deoxypentitol**	249377	0.580	0.054	0.347	1.982	NA
**uracil**	1174	-0.513	0.054	0.347	-1.980	C00106
**fucose**	439650	-0.314	0.055	0.347	-1.971	C02095
**myo-inositol**	892	0.306	0.060	0.358	1.937	C00137
**threitol**	169019	-0.564	0.064	0.358	-1.903	C16884
**glycyl-glycine**	11163	-0.340	0.064	0.358	-1.904	C02037
**valine**	6287	-0.314	0.066	0.358	-1.891	C00183
**cytidine**	6175	-0.547	0.073	0.384	-1.846	NA
**adenosine**	60961	0.654	0.076	0.387	1.823	C00212
**erythronic acid lactone isomer**	5325915	0.294	0.082	0.406	1.784	C02341
**glucose-6-phosphate**	5958	1.302	0.088	0.426	1.746	C00092
**cystine**	595	-0.693	0.092	0.431	-1.726	C01420
**creatinine**	588	0.197	0.097	0.441	1.696	C00791
**citric acid**	311	0.585	0.099	0.441	1.687	C00158
**glutaric acid**	743	0.495	0.103	0.447	1.670	C00489

**guanosine**	6802	0.505	0.109	0.448	1.637	C00387
**serotonin**	5202	-0.308	0.113	0.448	-1.620	C00780
**cysteine**	5862	-0.618	0.115	0.448	-1.609	C00097
**guanine**	764	-0.766	0.119	0.448	-1.591	C00242
**cholesterol**	5997	0.270	0.124	0.448	1.570	C00187
**methionine**	6137	-0.313	0.124	0.448	-1.570	C00073
**urea**	1176	0.389	0.125	0.448	1.564	C00086
**fructose**	439709	0.732	0.132	0.448	1.536	C02336
**uridine**	6029	-0.411	0.132	0.448	-1.535	C00299
**homocystine**	10010	-0.355	0.135	0.448	-1.526	C01817
**D-erythro-sphingosine**	5280335	-0.387	0.136	0.448	-1.521	C00319
**alanine**	5950	-0.330	0.137	0.448	-1.518	C00041
**ornithine**	6262	-0.282	0.140	0.448	-1.506	C00077
**hypoxanthine**	790	-0.175	0.141	0.448	-1.502	C00262
**levoglucosan**	2724705	-0.334	0.146	0.456	-1.485	C22350
**ribitol**	827	-0.395	0.150	0.462	-1.465	C00474
**aspartic acid**	5960	-0.272	0.155	0.469	-1.447	C00049
**asparagine**	6267	-0.194	0.161	0.473	-1.427	C00152
**azelaic acid**	19347555	0.330	0.165	0.473	1.415	C08261
**1-monostearin**	24699	0.428	0.169	0.473	1.402	D01947
**thymine**	1135	-0.307	0.172	0.473	-1.393	C00178
**palmitoleic acid**	445638	-0.311	0.175	0.473	-1.380	C08362
**citrulline**	9750	-0.401	0.176	0.473	-1.376	C00327
**methionine sulfoxide**	158980	-0.289	0.177	0.473	-1.372	C02989
**pseudo uridine**	15047	0.338	0.179	0.473	1.366	C02067
**galactinol**	11727586	0.311	0.195	0.503	1.318	NA
**methanolphosphate**	13130	0.257	0.197	0.503	1.310	NA
**glycerol-3-galactoside**	16048618	0.335	0.199	0.503	1.304	C05401
**octadecanol**	8221	0.492	0.205	0.509	1.289	NA
**erythrose**	439574	-0.240	0.211	0.513	-1.271	C01796
**N-acetyl-D-galactosamine**	440552	-0.322	0.213	0.513	-1.265	C05021
**beta-alanyl lysine**	440638	-0.453	0.217	0.513	-1.255	C05341
**2-aminobutyric acid**	6657	0.407	0.221	0.513	1.244	C02721
**pantothenic acid**	6613	-0.411	0.223	0.513	-1.238	C12276
**nicotinic acid MassBank**	938	0.194	0.225	0.513	1.233	C00253
**dihydrocholesterol**	66066	0.489	0.228	0.513	1.224	C12978
**glycerol-alpha-phosphate**	754	0.395	0.233	0.519	1.209	C03189
**pyrophosphate**	1023	0.318	0.237	0.521	1.199	C00013
**gluconic acid**	6857417	-0.427	0.246	0.533	-1.177	C00800
**sorbitol**	5780	0.355	0.255	0.546	1.153	C00794
**phosphate**	1004	0.169	0.260	0.546	1.142	C00009
**threonic acid**	5460407	0.282	0.262	0.546	1.138	C01620

**5'-deoxy-5'-methylthioadenosine**	439176	0.340	0.286	0.580	1.082	C00170
**tyrosine**	6057	-0.184	0.288	0.580	-1.076	C00082
**ethanolamine**	700	-0.187	0.288	0.580	-1.075	C00189
**fructose-6-phosphate**	440641	0.524	0.295	0.584	1.062	C05345
**N-acetylneuraminate MassBank**	439197	-0.179	0.297	0.584	-1.057	C00270
**cystathionine**	439258	0.380	0.310	0.602	1.029	C02291
**malic acid**	525	0.335	0.321	0.616	1.005	C00711
**tocopherol alpha-**	14985	0.309	0.346	0.657	0.953	C02477
**arachidic acid**	10467	-0.193	0.354	0.664	-0.939	C06425
**xanthine**	1188	-0.177	0.383	0.703	-0.881	C00385
**alpha-aminoadipic acid**	92136	0.275	0.386	0.703	0.876	C00956
**glycyl-proline**	3013625	-0.294	0.387	0.703	-0.875	NA
**N-acetylaspartate**	65065	0.163	0.392	0.706	0.864	C01042
**2-monoolein**	5319879	0.296	0.400	0.712	0.850	NA
**octadecylglycerol**	3681	-0.210	0.415	0.731	-0.823	C13858
**phenylalanine**	6140	-0.126	0.420	0.731	-0.815	C00079
**palmitic acid**	985	-0.108	0.424	0.731	-0.808	C00249
**2-hydroxyvaleric acid**	98009	0.164	0.438	0.747	0.784	NA
**behenic acid**	8215	-0.188	0.448	0.757	-0.767	C08281
**cysteine-glycine**	439498	-0.280	0.456	0.761	-0.752	C01419
**1-monopalmitin**	14900	-0.218	0.459	0.761	-0.747	NA
**putrescine**	1045	0.253	0.469	0.769	0.731	C00138
**ribonic acid**	5460677	-0.140	0.474	0.770	-0.723	C01685
**4-aminobutyric acid**	119	0.161	0.483	0.777	0.708	C00334
**4-hydroxyphenylacetic acid**	127	-0.245	0.493	0.786	-0.692	C00642
**beta-alanine**	239	-0.189	0.502	0.787	-0.677	C00099
**ribose**	5779	-0.121	0.506	0.787	-0.670	C00121
**beta-glycerolphosphate**	2526	-0.161	0.508	0.787	-0.668	C02979
**oleic acid**	445639	-0.124	0.528	0.806	-0.636	C00712
**glutamine**	5961	0.148	0.531	0.806	0.632	C00064
**inositol-4-monophosphate**	440043	0.219	0.539	0.806	0.620	C03546
**adenine**	190	0.178	0.539	0.806	0.620	C00147
**glucose-1-phosphate**	65533	-0.091	0.560	0.821	-0.587	C00103
**salicylic acid**	338	0.178	0.562	0.821	0.586	C00805
**gluconic acid lactone**	7027	-0.179	0.564	0.821	-0.582	C00198
**nicotinamide**	936	0.092	0.593	0.851	0.538	C00153
**sucrose**	5988	-0.185	0.594	0.851	-0.537	C00089
**tryptophan**	6305	-0.089	0.608	0.853	-0.517	C00078
**2-deoxytetronic acid**	192742	0.120	0.610	0.853	0.515	NA
**succinic acid**	1110	-0.143	0.611	0.853	-0.513	C00042
**xylitol**	6912	-0.132	0.620	0.854	-0.500	C00379

**glycine**	750	-0.055	0.622	0.854	-0.497	C00037
**cholesterone mino**	91477	0.117	0.647	0.881	0.462	C00599
**glyceric acid**	439194	0.070	0.655	0.885	0.451	C00258
**terephthalic acid**	7489	0.080	0.679	0.895	0.417	C06337
**3-hydroxy-3-methylglutaric acid**	1662	0.086	0.683	0.895	0.411	C03761
**UDP-glucuronic acid**	17473	0.104	0.683	0.895	0.411	C00167
**N-carbamylglutamate**	121396	-0.101	0.690	0.895	-0.401	C05829
**2,5-dihydroxypyrazine**	23368901	0.127	0.690	0.895	0.401	NA
**mannose**	18950	0.302	0.701	0.895	0.386	C00159
**trans-4-hydroxyproline**	5810	-0.073	0.702	0.895	-0.386	C01157
**oxoproline**	7405	0.038	0.705	0.895	0.382	C01879
**N-methyl-UMP**	44602426	0.157	0.718	0.900	0.364	NA
**glycolic acid**	757	0.063	0.719	0.900	0.363	C00160
**xylulose**	439205	0.066	0.727	0.904	0.351	C00312
**conduritol-beta-epoxide**	9989541	0.171	0.746	0.915	0.325	NA
**3-hydroxybutyric acid**	92135	0.114	0.749	0.915	0.322	C01089
**glucose**	64689	0.270	0.753	0.915	0.317	C00221
**hypotaurine**	107812	0.061	0.775	0.935	0.288	C00519
**3-phosphoglycerate**	724	0.103	0.788	0.941	0.270	C00597
**pentadecanoic acid**	13849	0.043	0.796	0.941	0.260	C16537
**diethanolamine**	8113	-0.162	0.798	0.941	-0.258	C06772
**caproic acid**	8892	-0.059	0.804	0.941	-0.249	C01585
**succinate semialdehyde**	9543238	0.057	0.808	0.941	0.245	NA
**lactic acid**	612	0.029	0.829	0.948	0.217	C01432
**heptadecanoic acid**	10465	0.031	0.833	0.948	0.212	NA
**butyrolactam**	12025	-0.061	0.838	0.948	-0.206	NA
**UDP-N-acetylglucosamine**	445675	0.042	0.839	0.948	0.205	C00043
**arachidonic acid**	444899	-0.032	0.841	0.948	-0.202	C00219
**alloxanoic acid**	94146	0.077	0.860	0.963	0.177	NA
**shikimic acid**	8742	-0.069	0.880	0.978	-0.152	C00493
**1,2-anhydro-myo-inositol**	119054	-0.051	0.899	0.982	-0.127	NA
**N-acetylglutamate**	70914	-0.023	0.902	0.982	-0.124	C00624
**adenosine-5-monophosphate**	6083	0.041	0.904	0.982	0.121	C00020
**linoleic acid**	5280450	-0.021	0.923	0.982	-0.098	C01595
**4-hydroxybutyric acid**	10413	0.025	0.923	0.982	0.097	C00989
**dihydroxyacetone**	670	0.047	0.929	0.982	0.089	C00184
**fumaric acid**	444972	0.017	0.940	0.982	0.076	C00122
**cis-gondoic acid**	5282768	0.014	0.943	0.982	0.072	C16526
**glutamic acid**	33032	-0.008	0.954	0.982	-0.058	C00025
**ile-ile**	7010568	-0.026	0.955	0.982	-0.057	C12H24N2O3
**lactose**	6134	-0.020	0.956	0.982	-0.056	C01970

**cysteine sulfonic acid**	129636156	-0.012	0.958	0.982	-0.053	NA
**MassBank**						
**spermidine**	1102	0.027	0.960	0.982	0.050	C00315
**xanthosine**	64959	0.007	0.972	0.982	0.035	C01762
**isothreonic acid**	151152	0.004	0.977	0.982	0.029	C00639
**adipic acid**	196	0.004	0.978	0.982	0.028	C06104
**2-hydroxyglutaric acid**	43	0.005	0.982	0.982	0.023	C02630

Table S3. Clinical and Pathological Measures

		Cases (n = 25)
**Age of onset**		
	N	20
	Mean (SD)	67.6 (8.9)
	Median (Range)	68.5 (50-81)
**Predominant symptom at onset**		
	Ataxia	11 (44%)
	Tremor	8 (32%)
	Cognitive	3 (12%)
	Neuropathy	1 (4%)
	Unknown	2 (8%)
**Tremor**		
	No tremor	4 (16%)
	Mild intention tremor	7 (28%)
	Intention tremor	4 (16%)
	Mixed tremor	7 (28%)
	Unknown	3 (12%)
**Ataxia**		
	No ataxia	1 (4%)
	Mild ataxia	3 (12%)
	Aid - Cane	1 (4%)
	Aid - Walker	6 (24%)
	Aid - Wheelchair	9 (36%)
	Bedridden	3 (12%)
	Unknown	2 (8%)
**Cognitive**		
	No cognitive impairment	3 (12%)
	Mild Cognitive Impairment	2 (8%)
	Dementia	11 (44%)

	Unknown	9 (36%)
**Parkinsonism**		
	No	10 (40%)
	Yes	12 (48%)
	Unknown	3 (12%)
**Neurological Clinical Co-diagnosis**		
	Alzheimer's Disease	3 (12%)
	Atypical Parkinsonian Syndrome	2 (8%)
	Mixed dementia	1 (4%)
	Parkinson's Disease	2 (8%)
	Stroke	1 (4%)
	Unknown	16 (64%)
**ITG Amyloid Plaques**		
	No	7 (28%)
	Yes	15 (60%)
	Not available	3 (12%)
**ITG Amyloid Plaques**		
	Sparse (<5)	4 (27%)
	Moderate (5 - 10)	6 (40%)
	Frequent (>10)	5 (33%)
**ITG CAA**		
	No	17 (68%)
	Yes	5 (20%)
	Not available	3 (12%)
**ITG Tau Tangles**		
	No	4 (16%)
	Yes	18 (72%)
	Not available	3 (12%)
**ITG Neuritic plaques**		
	No	11 (44%)

	Yes	11 (44%)
	Not available	3 (12%)
**ITG Neuritic plaques**		
	Sparse (<5)	11 (100%)
**ITG microhemorrhages**		
	No	11 (44%)
	Yes	11 (44%)
	Not available	3 (12%)

Figure S1 Relationship between metabolite abundance and CGG expansion

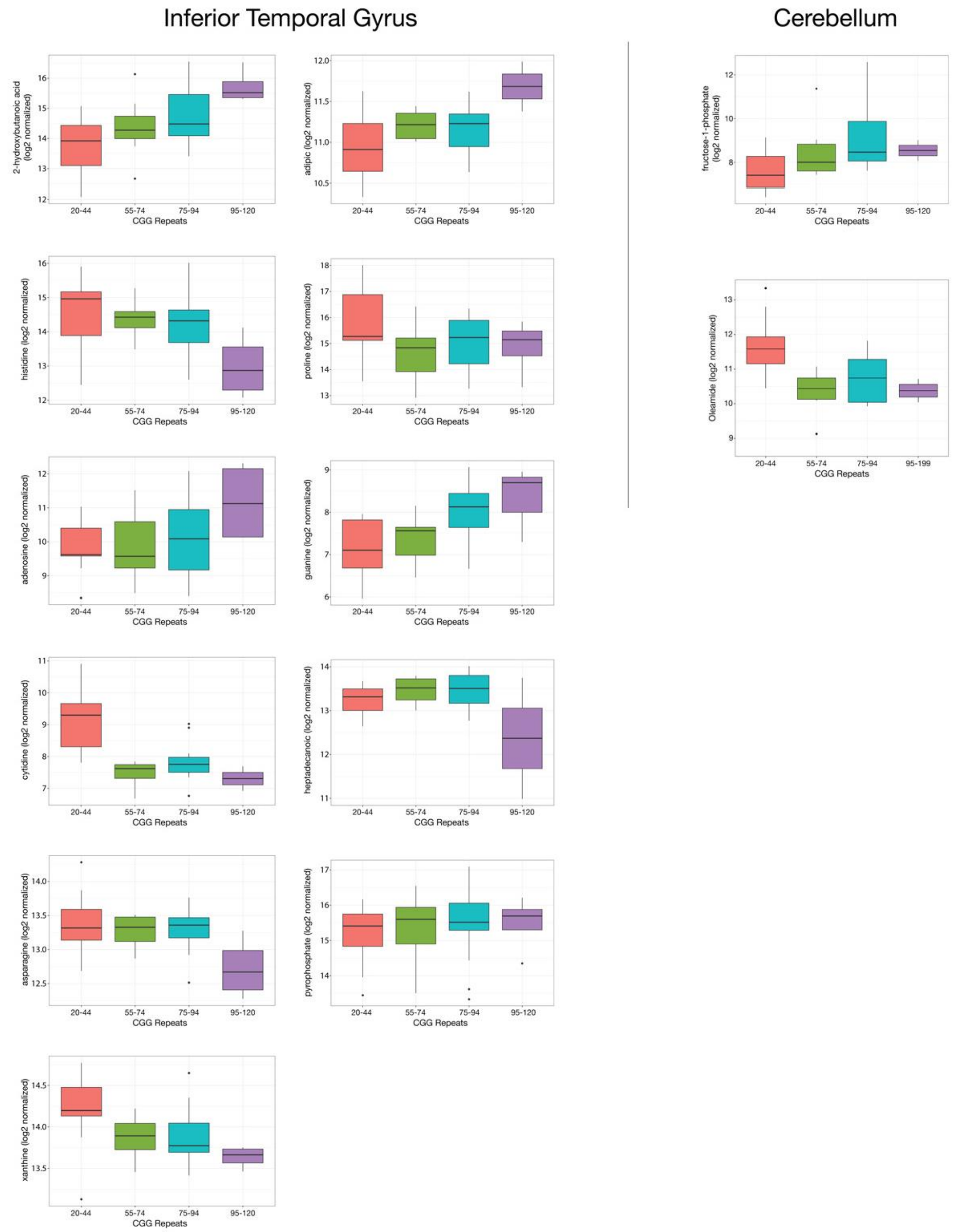

S1a) 2-HB acid had higher abundance in the ITG. Difference was trending (adj p-value 0.054) in the 95-120 CGG range group when compared to the normal CGG range expansion (20-44).
